# Autosomal Dominant Non-Syndromic Hearing Loss (DFNA): A Comprehensive Narrative Review

**DOI:** 10.3390/biomedicines11061616

**Published:** 2023-06-01

**Authors:** Mirko Aldè, Giovanna Cantarella, Diego Zanetti, Lorenzo Pignataro, Ignazio La Mantia, Luigi Maiolino, Salvatore Ferlito, Paola Di Mauro, Salvatore Cocuzza, Jérôme René Lechien, Giannicola Iannella, Francois Simon, Antonino Maniaci

**Affiliations:** 1 Department of Clinical Sciences and Community Health, University of Milan, 20090 Milan, Italy; mirko.alde@unimi.it (M.A.); giovanna.cantarella@unimi.it (G.C.); diego.zanetti.bs@gmail.com (D.Z.); lorenzo.pignataro@unimi.it (L.P.); 2 Department of Specialist Surgical Sciences, Fondazione IRCCS Ca’ Granda Ospedale Maggiore Policlinico, 20090 Milan, Italy; 3 Otology Study Group of the Young-Otolaryngologists of the International Federations of Oto-Rhino-Laryngological Societies (YO-IFOS), 75000 Paris, France; jerome.lechien@umons.ac.be (J.R.L.); giannicola.iannella@uniroma1.it (G.I.); f.simon@aphp.fr (F.S.); 4 Department of Medical, Surgical Sciences and Advanced Technologies G.F. Ingrassia, University of Catania, 95123 Catania, Italy; igolama@gmail.com (I.L.M.); maiolino@policlinico.unict.it (L.M.); ferlito@unict.it (S.F.); paola_mp86@hotmail.it (P.D.M.); s.cocuzza@unict.it (S.C.)

**Keywords:** genetic hearing loss, autosomal dominant inheritance, non-syndromic hearing loss, genes, loci

## Abstract

Autosomal dominant non-syndromic hearing loss (HL) typically occurs when only one dominant allele within the disease gene is sufficient to express the phenotype. Therefore, most patients diagnosed with autosomal dominant non-syndromic HL have a hearing-impaired parent, although de novo mutations should be considered in all cases of negative family history. To date, more than 50 genes and 80 loci have been identified for autosomal dominant non-syndromic HL. DFNA22 (*MYO6* gene), DFNA8/12 (*TECTA* gene), DFNA20/26 (*ACTG1* gene), DFNA6/14/38 (*WFS1* gene), DFNA15 (*POU4F3* gene), DFNA2A (*KCNQ4* gene), and DFNA10 (*EYA4* gene) are some of the most common forms of autosomal dominant non-syndromic HL. The characteristics of autosomal dominant non-syndromic HL are heterogenous. However, in most cases, HL tends to be bilateral, post-lingual in onset (childhood to early adulthood), high-frequency (sloping audiometric configuration), progressive, and variable in severity (mild to profound degree). DFNA1 (*DIAPH1* gene) and DFNA6/14/38 (*WFS1* gene) are the most common forms of autosomal dominant non-syndromic HL affecting low frequencies, while DFNA16 (unknown gene) is characterized by fluctuating HL. A long audiological follow-up is of paramount importance to identify hearing threshold deteriorations early and ensure prompt treatment with hearing aids or cochlear implants.

## 1. Introduction

The World Health Organization (WHO) estimates that approximately 34 million children worldwide have disabling hearing loss (HL), defined as HL greater than 35 dB in the better ear [1]. HL can be present at birth (“congenital HL”) or appear sometime later in life (“acquired or delayed-onset HL”) [2]. The prevalence of congenital sensorineural HL ranges from 1 to 3 per 1000 live births in term healthy newborns to 3–6 per 100 in children admitted to neonatal intensive care units (NICU) [3]. Overall, the prevalence of HL increases over time, ranging from 2.8 per 1000 in school-age children to 3.5 per 1000 in adolescents [4]. Non-hereditary HL can be caused by prenatal, perinatal, or postnatal factors. Prenatal risk factors for HL include prenatal exposure to teratogens (e.g., valproic acid, ethanol, and thalidomide), congenital infections (e.g., cytomegalovirus [CMV], toxoplasmosis, rubella, syphilis, and Zika), and malformations (e.g., Michel aplasia, enlarged vestibular aqueduct, Mondini malformation) [4,5].

Particularly, congenital CMV infection is considered the leading nongenetic cause of sensorineural HL in the developed world [6]. The characteristics of HL due to congenital CMV infection are extremely variable concerning onset (at birth/late onset), side (unilateral/bilateral), degree (mild/moderate/severe/profound), audiometric configuration (rising/flat/sloping), and threshold changes over time (stable, fluctuating, sudden, progressive) [6]. There are also several perinatal risk factors for HL, such as prematurity, very low birth weight, hyperbilirubinemia, asphyxia, and hypoxic-ischemic encephalopathy [3,5]. Postnatal risk factors for HL include infections (e.g., bacterial meningitis, Herpes Simplex Virus, and Epstein–Barr virus), use of ototoxic drugs (e.g., aminoglycosides, vancomycin, and furosemide), head trauma, chemotherapy, and anemia [5,7,8]. However, approximately 50–60% of HL in children is due to genetic causes, and a genetic etiology should be considered for every patient with a hearing problem, even in the presence of other environmental risk factors [9]. Hereditary HL can be syndromic (if other signs and symptoms are present) or non-syndromic (in the absence of other clinical manifestations) [7,10]. More than 70% of genetic HL is non-syndromic, with great clinical and genetic heterogeneity (more than 120 genes have been identified to date) [9,11]. Non-syndromic HL generally follows simple Mendelian inheritance and is predominantly transmitted as an autosomal recessive trait (75–80%), although autosomal dominant (20%), X-linked (2–5%), and mitochondrial mutations (1%) can also cause HL [12]. Children born to consanguineous parents have a higher incidence of autosomal recessive disorders, including HL [13]. The loci in inherited non-syndromic HL are designed as “DFN” (standing for “DeaFNess”); the letters “A”, “B”, and “X” indicate that the inheritance patterns are autosomal dominant (DFNA), autosomal recessive (DFNB), and X-linked (DFNX), respectively [7,10]. A Y-linked inheritance pattern has also been described for HL [14]. The most effective strategy for the diagnosis of non-syndromic genetic HL is to perform a multi-step approach based on next-generation sequencing technologies and copy number variations assays and a thorough clinical evaluation, including physical examination and audiometric tests [15]. The aim of this narrative review is to provide a comprehensive and critical overview of autosomal dominant non-syndromic genetic HL. We screened titles, abstracts, and full texts from relevant literature to evaluate the content of the articles and extract valuable information.

## 2. Autosomal Dominant Non-Syndromic Hearing Loss (DFNA)

### 2.1. Inheritance

Autosomal dominant inheritance occurs when only one dominant allele within the disease gene (located on one of the autosomal chromosomes) is sufficient to express the phenotype [16]. Therefore, a heterozygous parent with autosomal dominant non-syndromic HL (DFNA) has a 50% chance of passing it on to their children [7,16]. However, if one parent is homozygous, all offspring may inherit the disease. If both parents are heterozygous and affected by autosomal dominant non-syndromic HL, 75% of the offspring have the chance of inheriting the disease [16]. Males and females are equally likely to inherit the mutation [7,16]. Most patients diagnosed with autosomal dominant non-syndromic HL have a hearing-impaired parent [7]. However, although the family history is rarely negative, it may appear to be negative due to late-onset HL in a parent, reduced penetrance of the pathogenic variant in an asymptomatic parent, or a de novo variant [7]. In particular, de novo mutations are possible causes of genetic HL and should be considered in all cases of sporadic HL [17]. It is often difficult to distinguish between syndromic and non-syndromic HL, as symptoms can sometimes appear later. Furthermore, some genes (e.g., *WFS1* and *ACTG1*) cause both syndromic and non-syndromic HL [11].

To date, more than 50 genes and 80 loci have been identified for autosomal dominant non-syndromic HL [11], and are summarized in Table 1.

Unlike autosomal recessive non-syndromic HL (in which the majority of cases are caused by mutations in the *GJB2* gene), autosomal dominant non-syndromic HL does not have a single identifiable gene responsible for the majority of cases worldwide [7].

In Europe, the most common forms of autosomal dominant non-syndromic HL are DFNA22 (*MYO6* gene) and DFNA8/12 (*TECTA* gene), accounting for 21% and 18% of all cases, respectively [126]. Other frequent forms of autosomal dominant non-syndromic HL in Europe are DFNA20/26 (*ACTG1* gene), DFNA6/14/38 (*WFS1* gene), and DFNA15 (*POU4F3* gene), accounting for 9%, 9%, and 6.5% of all cases, respectively [126]. *KCNQ4* (DFNA2A) and *EYA4* (DFNA10) genes contribute 2.5% each, while the remaining genes are residually represented [126]. De novo mutations have been described in several genes, such as *GJB2* (DFNA3A) [127,128], *ACTG1* (DFNA20/26) [129,130], *TECTA* (DFNA8/12) [131], *MYH14* (DFNA4A) [131], *CEACAM16* (DFNA4B) [132], *ATP2B2* (DFNA82) [118], and *WFS1* (DFNA6/14/38) [133].

### 2.2. MYO6 Gene

Mutations in the *MYO6* gene can cause either autosomal dominant non-syndromic HL (DFNA22) or autosomal recessive non-syndromic HL (DFNB37) [11]. DFNA22 is caused by a heterozygous mutation in the myosin VI gene (*MYO6*) on chromosome 6q14 [11]. Myosin VI is an actin-based motor protein which plays a key role in the endocytic and exocytic membrane trafficking pathways. In the inner and outer hair cells of the organ of Corti, myosin VI serves as an anchor and maintains the structure of the stereocilia [134]. Autosomal dominant HL associated with *MYO6* mutations was reported in large Italian [135], Danish [136], Belgian [53,137], Dutch [138], German [139], and Austrian [140] families. However, several cases of DFNA22 were described in China [141,142,143], Japan [144,145], the Republic of Korea [146], and Brazil [147]. HL is typically post-lingual (often occurs during childhood), is slowly progressive, ranges from a mild to profound degree, and may be associated with mild cardiac hypertrophy [11,148]. Volk et al. suggested a favorable outcome of cochlear implantation in patients with DFNA22 [139].

### 2.3. TECTA Gene

Autosomal dominant non-syndromic sensorineural deafness 8/12 (DFNA8/12) is caused by heterozygous mutations in the *TECTA* gene on chromosome 11q23 [11]. Missense mutations of *TECTA* cause DFNA8/12, while nonsense mutations cause autosomal recessive non-syndromic HL (DFNB21) [11]. The *TECTA* gene encodes alpha-tectorin, one of the major non-collagenous components of the tectorial membrane of the inner ear that bridges the stereocilia bundles of the sensory hair cells [35]. HL associated with *TECTA* missense mutations was reported in families from different European countries, including Belgium [149,150], Austria [151,152], France [153], Sweden [154], Spain [155], and The Netherlands [156,157,158,159]. However, autosomal dominant non-syndromic HL caused by TECTA mutations was also reported in Japanese [160,161,162,163], Turkish [164], American [35,165], Korean [166,167], Brazilian [168], Chinese [141,169,170], Mongolian [171], and Algerian [172] families. HL can be present before the child learns to speak (prelingual) or begin in childhood (first or second decade of life). The characteristics of HL depend on the domain in which the mutations occur: missense mutations in the *zona pellucida* domain lead to mid-frequency sensorineural HL (“U-shaped” or “cookie bite” audiometric configuration), while missense mutations in the *zonadhesin region* cause high-frequency sensorineural HL (“sloping” audiometric configuration). HL is progressive if cysteine residues are affected [11,35].

### 2.4. ACTG1 Gene

Autosomal dominant non-syndromic sensorineural deafness 20/26 (DFNA20/26) is caused by heterozygous mutations in the *ACTG1* gene on chromosome 17q25 [11]. Mutations in the *ACTG1* gene can be associated with autosomal dominant non-syndromic HL (DFNA20/26) and Baraitser–Winter syndrome (a rare condition characterized by ptosis, colobomata, neuronal migration disorder, distinct facial anomalies, and intellectual disability) [11,173]. The *ACTG1* gene encodes gamma actin, which is a major actin protein in the cytoskeleton of auditory hair cells and is essential for the maintenance of stereocilia [173]. In Europe, DFNA20/26 was reported in Dutch [51,174,175], Norwegian [176], Spanish [177], and Italian [173] families. Mutations in the *ACTG1* gene were also frequently described in American [50,178,179,180], Chinese [129,181,182,183,184], Korean [185,186,187], and Japanese [188,189,190] populations. HL is typically diagnosed in the first or second decade of life and affects high frequencies (“sloping” audiometric configuration). It is progressive and tends to become profound by the sixth decade of life [11].

### 2.5. WFS1 Gene

Autosomal dominant non-syndromic sensorineural deafness 6/14/38 (DFNA6/14/38) is caused by heterozygous mutations in the *WFS1* gene on chromosome 4p16 [11]. The DFNA6, DFNA14, and DFNA38 loci were initially described separately but were later found to be associated with pathogenic variants in the same gene (*WFS1*) [191]. Mutations in the *WSF1* gene can be responsible for both autosomal dominant non-syndromic HL (DFNA6/14/38) and Wolfram syndrome (an autosomal recessive disorder characterized by diabetes mellitus, diabetes insipidus, optic atrophy, and high-frequency sensorineural HL) [11,191]. The *WFS1* gene encodes “Wolframin”, a transmembrane protein located in the endoplasmic reticulum and ubiquitously expressed [191]. DFNA6/14/38 was largely described in the United States of America [192,193,194,195,196,197], Japan [198,199,200,201,202,203], and China [133,182,204,205,206,207,208,209,210]. In Europe, DFNA6/14/38 was reported in Dutch [191,211,212,213], Swiss [214], Danish [215], Hungarian [216], Finnish [217], and German [218] families. Other cases of DFNA6/14/38 were observed in Taiwan [219], the Republic of Korea [220,221], Iran [222], and India [223]. HL is generally congenital, limited to low frequencies (2000 Hz and below), and slowly progressive (without reaching a severe-to-profound range). It may be associated with tinnitus, but speech perception is typically good [11]. Interestingly, although Wolframin is equally expressed in the basal and apical turns of the cochlea, HL involves the low frequencies in DFNA6/14/38 and the high frequencies in Wolfram syndrome [191]. 

### 2.6. POU4F3 Gene

Autosomal dominant non-syndromic sensorineural deafness 15 (DFNA15) is caused by heterozygous mutations in the *POU4F3* gene on chromosome 5q32 [11]. The *POU4F3* gene encodes a transcription factor which plays a key role in the maintenance of inner ear hair cells [224]. DFNA15 was largely described in Israeli [44,225,226,227] and Chinese families [45,141,182,228,229,230,231,232]. In Europe, DFNA15 was widely reported in The Netherlands [233,234,235,236]. Other cases of DFNA15 were observed in the Republic of Korea [185,237,238], Brazil [239,240], Japan [188,241], and Taiwan [242]. HL is post-lingual (onset varies between the second and sixth decades of life), bilateral, and progressive [11]. It is characterized by high intrafamilial variability and tends to progress to the severe-to-profound range. Audiometric configuration can be sloping or flat [11]. HL may also be associated with vestibular dysfunctions, including areflexia [243].

### 2.7. KCNQ4 Gene

Autosomal dominant non-syndromic sensorineural deafness 2A (DFNA2A) is caused by a heterozygous mutation in the *KCNQ4* gene on chromosome 1p34.2 [11].

The protein encoded by the *KCNQ4* gene forms a potassium channel that plays a key role in the regulation of neuronal excitability, particularly in the sensory cells of the cochlea [244]. Autosomal dominant HL due to *KCNQ4* mutations was reported in Indonesian [245], American [246,247,248,249,250], Japanese [251,252,253,254], Taiwanese [255,256,257], Canadian [22], Brazilian [258], Pakistani [259], Iranian [260], Chinese [261,262,263,264], and Korean [265,266,267,268] families. In Europe, DFNA2A was observed in French [247,269], Dutch [247,252,270,271,272,273,274], Belgian [247,271], and Spanish [275] families. HL is generally diagnosed between 5 and 15 years old and is initially limited to high frequencies, with later involvement of the middle and high frequencies. It tends to be severe by age 50 [11]. Most patients had associated tinnitus but no vestibular symptoms except in a few cases [254].

### 2.8. EYA4 Gene

Autosomal dominant non-syndromic sensorineural deafness 10 (DFNA10) is caused by heterozygous mutations in the *EYA4* gene on chromosome 6q23 [11]. The *EYA4* gene encodes a member of the eyes absent (EYA) family of proteins, which is a transcriptional activator required for proper eye development as well as for the maturation and maintenance of the organ of Corti. Mutations in *EYA4* can also cause a syndromic variant characterized by HL and dilated cardiomyopathy [276]. DFNA10 was observed in large American [277,278,279,280,281], Australian [38], Indian [223], Korean [282,283,284], Chinese [276,285,286,287,288,289,290], Brazilian [240], and Japanese [39,291] families. In Europe, HL due to mutations in the *EYA4* gene were reported in Belgian [278,280,292,293], Norwegian [292], Hungarian [294], Swedish [295], Dutch [296], Italian [297], Slovakian [298], and Spanish [299] families. HL is typically progressive and often involves all frequencies, although initially, it may be limited to middle frequencies. The onset of HL is highly variable [11]. The audiometric configuration of truncating variants tends to be flat, while that of non-truncating variants tends to be sloping [39]. DFNA10 patients are considered the least responsive to cochlear implantation [38].

### 2.9. Characteristics of Hearing Loss

The characteristics of autosomal dominant non-syndromic HL are heterogenous. Most autosomal dominant loci cause post-lingual HL, with onset ranging from childhood to late adulthood (Table 1). However, HL tends to occur in childhood, adolescence, or early adulthood. Moreover, a non-negligible number of loci are associated with congenital HL, including DFNA3A (*GJB2* gene), DFNA3B (*GJB6* gene), DFNA6/14/38 (*WFS1* gene), DFNA7 (*LMX1A* gene), DFNA8/12 (*TECTA* gene), DFNA13 (*COL11A2* gene), DFNA19 (unknown gene), DFNA23 (*SIX1* gene), DFNA24 (unknown gene), DFNA27 (*REST* gene), DFNA30 (unknown gene), DFNA37 (*COL11A1* gene), DFNA40 (*CRYM* gene), DFNA59 (unknown gene), DFNA66 (*CD164* gene), DFNA69 (*KITLG* gene), DFNA71 (*DMXL2* gene), DFNA78 (*SLC12A2* gene), DFNA80 (*GREB1L* gene), DFNA84 (*ATP11A* gene), DFNA87 (*PI4KB* gene), and DNA89 (*ATOH1* gene) (Table 1). The degree of HL at onset ranges from mild to profound. Most cases of HL are progressive and worsen over the years, with the exceptions of DFNA8/12 (*TECTA* gene), DFNA13 (*COL11A2* gene), DFNA19 (unknown gene), DFNA23 (*SIX1* gene), DFNA24 (unknown gene), DFNA40 (*CRYM* gene), DFNA59 (unknown gene), DFNA66 (*CD164* gene), DFNA69 (*KITLG* gene), DFNA76 (*PLS1* gene), DFNA78 (*SLC12A2* gene), and DFNA80 (*GREB1L* gene), which tend to be stable (Table 1). Interestingly, DFNA16 (unknown gene) is characterized by fluctuating HL that often benefits from treatment with oral steroids [46]. Audiometric configuration is highly variable, although it often tends to be sloping, with the high frequencies more involved, especially at the onset of HL. A flat audiometric configuration is also frequent (Table 1). However, some loci are associated with rising audiometric configuration, and HL is limited to the low frequencies: DFNA1 (*DIAPH1* gene), DFNA6/14/38 (*WFS1* gene), DFNA44 (*CCDC50* gene), DFNA49 (unknown gene), DFNA54 (unknown gene), DFNA56 (*TNC* gene), DFNA57 (unknown gene), and sometimes DFNA11 (*MYO7A* gene), and DFNA69 (*KITLG* gene) (Table 1).

DFNA1 and DFNA6/14/38 are the most common forms of autosomal dominant non-syndromic HL affecting the low frequencies. DFNA1 is due to mutations in the *DIAPH1* gene on chromosome 5q31 and causes progressive low-frequency HL, resulting in a profound degree by the fourth decade of life [18,19]; conversely, DFNA6/14/38 is due to mutations in the *WFS1* gene on chromosome 4p16 and does not progress to profound HL [33]. The “U-shaped”, “saucer”, or “cookie bite” audiometric configuration indicates mid-range frequency HL and can be associated with some autosomal dominant loci, including DFNA8/12 (*TECTA* gene), DFNA13 (*COL11A2* gene), DFNA31 (unknown gene), DFNA37 (*COL11A1* gene), DFNA66 (*CD164* gene), and DFNA72 (*SLC44A4* gene) (Table 1). Although non-syndromic HL is typically not associated with other clinical manifestations, some autosomal dominant loci can cause other signs or symptoms than HL, such as thrombocytopenia (DFNA1), vertigo or vestibular dysfunction (DFNA7, DFNA9, DFNA11, DFNA15, DFNA16, DFNA36, DFNA54, DFNA69, DFNA78, and DFNA82), cochleosaccular dysplasia (DFNA17), hypertrophic cardiomyopathy (DFNA22), preauricular pits, hypodysplastic kidney, and vesicoureteral reflux (DFNA23), autoinflammatory disorders (DFNA34), dentinogenesis imperfecta (DFNA39), absent or malformed cochleae and eighth cranial nerves (DFNA80), and incomplete cochlea partition and enlarged vestibular aqueduct (DFNA87) (Table 1).

### 2.10. How Knowledge of Genetic Mutations May Influence Treatment

All children diagnosed with sensorineural HL should be screened early for genetic mutations to ensure timely appropriate treatments (e.g., hearing aid or cochlear implant), personalized rehabilitation programs (e.g., in the presence of additional symptoms), prognosis (e.g., stable, progressive, or fluctuant HL), and family planning [7]. The team evaluating and treating these children should consist of an otolaryngologist with expertise in the management of pediatric otologic disorders, an audiologist experienced in the assessment of childhood HL, a clinical geneticist, a speech-language pathologist specializing in working with children affected by HL, and a pediatrician [7]. For children with severe-to-profound HL, hearing aids may be insufficient for HL rehabilitation, and cochlear implantation should be considered.

Cochlear implantation has a high probability of being effective if the mutated lesion is located in the hair cells or afferent synapses between hair cells and the auditory nerve, such as in patients with pathogenic variants in *GJB2*, *COCH*, *MYO7A*, *ACTG1*, or *MYO6* genes. Conversely, cochlear implants are generally less effective if genetic mutations affect auditory nerve function [139,300,301,302,303]. Moreover, genetic testing is useful not only for predicting performance after cochlear implantation but also for assessing residual hearing, estimating progression, and successful hearing preservation, leading to the most appropriate selection of candidates and electrodes [302].

As a matter of fact, better knowledge regarding genotype–phenotype correlation and cochlear implant outcome may provide effective auditory rehabilitation and would reduce unnecessary procedures, thereby limiting both surgical risks and healthcare costs [303].

### 2.11. Current Limitations and Future Trends

The genetics of non-syndromic HL are constantly evolving, and there are currently many limitations of knowledge in this field. The etiology of some patients with evident familial HL still remains unknown. Indeed, intra-familial variability in sensorineural HL is common not only from parent to child in dominant cases but also between siblings [12]. Many pathogenic variants affecting known deafness genes may go undetected using current diagnostic algorithms because they reside in non-coding (intronic and regulatory) sequences or unannotated exons [304]. Therefore, consideration should be given to implementing whole exome or whole genome sequencing with a virtual panel as the gold standard for genetic testing in HL instead of targeted gene sequencing panels [305].

Currently, many children with mild or progressive forms of HL remain undiagnosed during their critical period of speech development and neuroplasticity. Therefore, it appears to be a priority to develop a new cost-effective method of universal genetic screening that ensures early diagnosis of genetic HL in order to identify potential comorbid conditions and guide treatments [306].

In recent years, there have been major advances in the development of gene therapy vectors to treat sensorineural HL in animal models, representing a promising approach to prevent or slow down genetic HL. Interestingly, gene therapy is not limited to the addition of a healthy copy of the defective gene but may also involve gene silencing or editing through nucleic acid-based strategies, including antisense oligonucleotides, siRNA, microRNA, or nuclease-based gene editing [307]. However, many issues are still unresolved, such as the temporal window for therapeutic intervention, the need for viral vector optimization, the safety of surgery, and the type of immune response [308].

## 3. Conclusions

Patients diagnosed with autosomal dominant non-syndromic HL typically have a parent affected by HL, although de novo mutations should be considered in the case of negative family history. Overall, autosomal dominant non-syndromic HL tends to be bilateral, post-lingual in onset, high-frequency, progressive, and variable in severity. However, congenital, low-frequency, and stable forms of HL are also possible. A long and accurate audiological follow-up is of paramount importance to early identify hearing threshold deterioration and ensure prompt treatment with hearing aids or cochlear implants according to the degree of HL.

Despite the importance of the major findings, the study has many limitations, including that it does not show the mutations described in each gene and whether there are “hot spots” mutations or domains in which these mutations are localized.

## Figures and Tables

**Table 1:** Autosomal dominant non-syndromic hearing loss: loci, genes and clinical manifestations.

Locus	Cytogenetic Location	Gene	Protein Function	HL Onset	Audiometric Configuration	HL Trend	Comments
DFNA1	5q31.3	*DIAPH1*	Cytoskeletal organization of inner ear hair cells.	Childhood (1st decade)	Rising	Progressive	HL may be associated with thrombocytopenia or auditory neuropathy and tends to progress to a profound degree by the 4th decade of life [18,19,20].
DFNA2A	1p34.2	*KCNQ4*	Potassium channel in the cochlear sensory cells.	Childhood/Adolescence (1st–2nd decade)	Sloping	Progressive	At younger ages, HL is mild in the low frequencies and moderate in the high frequencies. Over the years, hearing progressively deteriorates in all frequencies [21,22].
DFNA2B	1p34.3	*GJB3*	Gap junction (connexin 31).	Adulthood (4th decade)	Sloping	Progressive	HL tends to be milder in females [23,24].
DFNA2C	1p36.11	*IFNLR1*	Cytokine receptor.	Adulthood (3rd-4th decade)	Sloping	Progressive	Hearing is initially normal in the low frequencies, but it progressively deteriorates in all frequencies [25].
DFNA3A	13q12.11	*GJB2*	Gap junction (connexin 26).	Congenital/Childhood (1st decade)	Sloping	Progressive	The degree of HL can range from mild to profound [26].
DFNA3B	13q12.11	*GJB6*	Gap junction (connexin 30).	Congenital/Childhood (1st decade)	Sloping	Progressive	The degree of HL can range from mild to profound [27].
DFNA4A	19q13.33	*MYH14*	Regulation of cytokinesis, cell motility, and polarity.	Childhood to adulthood (1st–3rd decade)	Flat	Progressive	The initial audiogram may be slightly sloping or U-shaped, but it becomes flat over the years [28,29].
DFNA4B	19q13.31–q13.32	*CEACAM16*	Connection between the outer hair cells stereocilia and tectorial membrane.	Childhood/Adolescence (1st–2nd decade)	Flat	Progressive	HL may initially be limited to high frequencies but progressively involves all frequencies [30].
DFNA5	7p15.3	*GSDME*	Regulation of apoptosis.	Childhood to adulthood (1st–6th decade)	Sloping	Progressive	HL may initially be limited to high frequencies but progressively involves all frequencies [31,32].
DFNA6/14/38	4p16.1	*WFS1*	Cation-selective ion channel.	Congenital to adulthood (1st–3rd decade)	Rising	Progressive	Hearing worsens over time but does not progress to profound HL [33].
DFNA7	1q23.3	*LMX1A*	Transcription factor.	Congenital to adulthood (1st–6th decade)	Sloping	Progressive	HL is characterized by high variability in age of onset and severity. HL can be associated with vertigo [34].
DFNA8/12	11q23.3	*TECTA*	Non-collagenous component of the tectorial membrane.	Congenital/Childhood (1st–2nd decade)	U-shaped/ Sloping	Stable/ Progressive	Missense mutations in the *zona pellucida* domain of TECTA cause moderate HL in the middle frequencies, while missense mutations in the *zonadhesin* region cause mild-to- moderate HL in the high frequencies. HL is progressive if cysteine residues are affected [35].
DFNA9	14q12	*COCH*	Structural support to the cochlea and interaction with other molecules in the extracellular matrix.	Adolescence/Adulthood (2nd–3rd decade)	Sloping	Progressive	HL is associated with variable vestibular dysfunction and tends to progress to anacusis by the 5th decade of life [36,37].
DFNA10	6q23.2	*EYA4*	Transcriptional activator.	Adolescence/Adulthood (1st–5th decade)	Flat/Gently sloping	Progressive	Truncating variants tend to cause flat-type HL that deteriorates at all frequencies, while non-truncating variants tend to cause high-frequency HL [38,39].
DFNA11	11q13.5	*MYO7A*	Unconventional myosin that serves in intracellular movements.	Childhood to adulthood (1st–5th decade)	Flat/Gently sloping/Rising	Progressive	HL may be associated with mild vestibular dysfunctions and is gradually progressive [40,41].
DFNA13	6p21.32	*COL11A2*	Fibril-forming collagen found mainly in the cartilage extracellular matrix.	Congenital to adulthood (1st–4th decade)	U-shaped	Stable	HL is generally non-progressive and limited to middle frequencies [42,43].
DFNA15	5q32	*POU4F3*	Transcription factor.	Adolescence/Adulthood (2nd–6th decade)	Sloping/Flat	Progressive	HL may be associated with vestibular dysfunction and is characterized by intrafamilial variability. It tends to progress to a profound degree over the years [44,45].
DFNA16	2q23–q24.3	Unknown	Unknown.	Childhood (1st decade)	Sloping	Fluctuating	HL may be associated with vertigo. In women, hearing may worsen immediately after delivery. Treatment with oral steroids can restore hearing during episodes of acute HL [46].
DFNA17	22q12.3	*MYH9*	Homeostasis of the organ of Corti, spiral ligament, and Reissner membrane.	Childhood to adulthood (1st–5th decade)	Sloping	Progressive	HL is associated with cochleosaccular dysplasia and organ of Corti degeneration [47].
DFNA18	3q22	Unknown	Unknown.	Childhood (1st decade)	Sloping	Progressive	HL initially involves only the high frequencies, but over the years, it also affects the middle and low frequencies [48].
DFNA19	10 centromic	Unknown	Unknown.	Congenital	Flat	Stable	HL is mild-to-moderate and non-progressive [49].
DFNA20/26	17q25.3	*ACTG1*	Cytoskeletal organization of inner ear hair cells and stereocilia maintenance.	Childhood/Adolescence (1st–2nd decade)	Sloping	Progressive	HL tends to progress to a profound degree by the 6th decade of life [50,51].
DFNA21	6p24.1–p22.3	*RIPOR2*	Essential component of hair cell stereocilia.	Childhood to adulthood (1st–5th decade)	Sloping	Progressive	HL is gradually sloping and progressive [52].
DFNA22	6q14.1	*MYO6*	Manteinance of hair cell stereocilia.	Childhood to adulthood (1st–3rd decade)	Sloping/Flat	Progressive	HL may be associated with mild hypertrophic cardiomyopathy. It tends to progress to a profound degree by the 5th decade of life [53].
DFNA23	14q23.1	*SIX1*	Control of genes involved in ear development.	Congenital	Sloping	Stable	HL is generally non-progressive and may be associated with preauricular pits, hypodysplastic kidneys, and vesicoureteral reflux [54].
DFNA24	4q35-qter	Unknown	Unknown.	Congenital	Sloping	Stable	The degree of HL can range from mild to profound [55].
DFNA25	12q23.1	*SLC17A8*	Vesicular glutamate transporter.	Childhood to adulthood (1st–6th decade)	Sloping	Progressive	HL is slowly progressive [56].
DFNA27	4q12	*REST*	Transcriptional repressor.	Congenital to adulthood (1st–3rd decade)	Flat	Progressive	HL tends to progress to a profound degree by the 5th decade of life [57,58].
DFNA28	8q22.3	*GRHL2*	Transcription factor.	Childhood (1st decade)	Flat/Gently Sloping	Progressive	HL tends to progress to a severe degree at higher frequencies by the 5th decade [59].
DFNA30	15q25-q26	Unknown	Unknown.	Congenital to adulthood (1st–4th decade)	Sloping	Progressive	HL is initially limited to high frequencies but progressively involves the middle frequencies [60].
DFNA31	6p21.3	Unknown	Unknown.	Childhood to adulthood (1st–4th decade)	U-shaped/Flat	Progressive	HL is characterized by high variability in age of onset, audiometric configuration, and progression [61].
DFNA32	11p15	Unknown	Unknown.	/	/	Progressive	This locus was reported only as an abstract [62].
DFNA33	13q34-qter	Unknown	Unknown.	Adolescence/Adulthood (2nd–3rd decade)	Sloping	Progressive	HL is initially limited to high frequencies but progressively involves all frequencies [63].
DFNA34	1q44	*NLRP3*	Critical component of the NLRP3 inflammasome that is activated in innate immune responses.	Childhood to adulthood (1st–4th decade)	Sloping	Progressive	HL is slowing progressive and may be associated with autoinflammatory disorders (e.g., oral ulcers, arthralgia, arthritis, urticaria, periodic fever, and lymphadenopathy) [64].
DFNA36	9q21.13	*TMC1*	Component of mechanotransduction channels in hair cells of the inner ear.	Childhood to adulthood (1st–3rd decade)	Sloping/Flat	Progressive	HL tends rapidly to involve all frequencies and progress to a profound degree. It may be associated with vertigo [65,66].
DFNA37	1p21.1	*COL11A1*	Essential for skeletal, ocular and auditory functions.	Congenital/Childhood (1st decade)	U-shaped/Flat/Gently sloping	Progressive	HL is generally in the mild-to-moderate range and tends to a slow progression [67].
DFNA39	4q22.1	*DSPP*	Dentin mineralization and inner ear homeostasis.	Adulthood (3rd decade)	Sloping	Progressive	HL is associated with dentinogenesis imperfecta [68].
DFNA40	16p12.2	*CRYM*	Thyroid hormone binding for possible regulatory roles.	Congenital/Childhood (1st decade)	Sloping	Progressive/ Stable	HL is generally in the moderate-to-severe range [69].
DFNA41	12q24.33	*P2RX2*	Ligand-gated ion channel.	Childhood/Adolescence (1st–2nd decade)	Sloping	Progressive	HL is exacerbated by noise exposure and tends to be severe by the 3rd decade of life [70,71].
DFNA42/52	5q31.1–q32	Unknown	Unknown.	Adulthood (2nd–3rd decade)	Sloping	Progressive	HL is initially limited to high frequencies but progressively involves all frequencies, resulting in profound HL [72,73].
DFNA43	2p12	Unknown	Unknown.	Adulthood (2nd–3rd decade)	Sloping	Progressive	HL is slowly progressive, extending to all frequencies by the 5th/6th decade of life [74].
DFNA44	3q28	*CCDC50*	Effector of epidermal growth factor-mediated cell signaling.	Childhood (1st decade)	Rising	Progressive	HL is initially limited to low and mild frequencies but gradually involves all frequencies, progressing to profound HL in the 6th decade of life [75].
DFNA47	9p21–p22	Unknown	Unknown.	Adulthood (2nd–3rd decade)	Sloping	Progressive	HL is initially limited to high frequencies but progressively involves all frequencies, reaching the moderate-to-severe range by the 5th decade of life [76].
DFNA48	12q13.3–q14	*MYO1A*	Unconventional myosin.	Childhood to adulthood (1st–3rd decade)	Flat	Progressive	HL is slowly progressive. The degree of HL can range from moderate to severe [77,78].
DFNA49	1q21–q23	Unknown	Unknown.	Childhood (1st decade)	Rising	Progressive	HL is initially limited to low and middle frequencies. By the 4th decade of life, audiometric configuration reaches a U shape (severe HL for middle frequencies and moderate HL for low and high frequencies) [79].
DFNA50	7q32.2	*MIR96*	Essential for differentiation and function of the inner ear.	Adolescence (2nd decade)	Flat	Progressive	HL is initially mild and progresses to a severe-to-profound range by the 7th decade of life [80,81].
DFNA51	9q21.11	*TJP2*	Organization of epithelial and endothelial intercellular junctions.	Adulthood (4th decade)	Sloping	Progressive	HL progressively involves all frequencies, resulting in profound HL [82].
DFNA53	14q11.2–q12	Unknown	Unknown.	Adolescence (2nd decade)	Sloping	Progressive	HL is initially mild and limited to high frequencies but gradually involves all frequencies and progresses to a profound degree by the 4th/5th decade of life [83].
DFNA54	5q31	Unknown	Unknown.	Childhood to adulthood (1st–3rd decade)	Rising	Progressive	HL slowly progresses to a severe degree and may be associated with vertigo [84].
DFNA55	9p13.2–p13.3	Unknown	Unknown.	/	/	/	This locus was reported only in a Chinese journal [85].
DFNA56	9q33.1	*TNC*	Guidance of migrating neurons during development.	Childhood to adulthood (1st-3rd decade)	Rising	Progressive	HL is initially mild and limited to low frequencies but gradually involves all frequencies, progressing to a severe degree [86].
DFNA57	19p13.2	Unknown	Unknown.	Childhood (1st decade)	Rising	Progressive	HL is initially limited to low frequencies but gradually involves all frequencies and progresses to the moderate-to-severe range by the 5th/6th decade of life [87].
DFNA58	2p12–p21	Unknown	Unknown.	Adolescence/Adulthood (2nd–4th decade)	Sloping	Progressive	HL is initially mild and limited to high frequencies but gradually involves all frequencies, progressing to a severe degree [88].
DFNA59	11p14.2–q12.3	Unknown	Unknown.	Congenital	Sloping	Stable	HL is severe-to-profound and non-progressive [89].
DFNA60	2q21.3–q24.1	Unknown	Unknown.	Adolescence/Adulthood (2nd–3rd decade)	/	Progressive	This locus was reported only as an abstract [90].
DFNA63	3q25.1–q25.2	Unknown	Unknown.	/	/	/	This locus was assigned by the HUGO nomenclature committee, but no information is available [91].
DFNA64	12q24.31	*DIABLO*	Regulation of apoptosis.	Adolescence/Adulthood (2nd–3rd decade)	Flat	Progressive	High-frequency tinnitus is often present at the onset of HL [92].
DFNA65	16p13.3	*TBC1D24*	Regulation of membrane trafficking.	Adulthood (3rd decade)	Sloping	Progressive	HL is initially limited to high frequencies, but slowly progresses to all frequencies, reaching the severe-to-profound range in the 7th decade of life [93,94].
DFNA66	6q15–21	*CD164*	Transmembrane sialomucin and cell adhesion molecule.	Congenital to adulthood (1st–3rd decade)	Flat/U-shaped	Stable/ Progressive	HL is characterized by high variability in the age of onset and progression [95].
DFNA67	20q13.33	*OSBPL2*	Intracellular lipid receptor.	Childhood to adulthood (1st–4th decade)	Sloping	Progressive	HL is initially limited to high frequencies but rapidly progresses to all frequencies [96].
DFNA68	15q25.2	*HOMER2*	Intracellular calcium homeostasis and cytoskeletal organization.	Childhood/Adolescence (1st–2nd decade)	Sloping	Progressive	HL is initially limited to high frequencies but gradually progresses to all frequencies [97,98].
DFNA69	12q21.32	*KITLG*	Ligand of the tyrosine-kinase receptor.	Congenital	Flat/Sloping/ Rising	Stable	HL is unilateral or bilateral asymmetric. HL may be associated with subclinical vestibular dysfunctions [99].
DFNA70	3q21.3	*MCM2*	Important role in the onset of DNA replication and cell division.	Adolescence/Adulthood (≥2nd decade)	Sloping/Flat	Progressive	HL is slowly progressive, resulting in a mild to profound degree [100,101].
DFNA71	15q21.2	*DMXL2*	Participation in signal transduction pathways.	Congenital to adolescence (1st–2nd decade)	Flat	Progressive	HL gradually progresses to a severe-to-profound degree in the 5th decade of life [102,103].
DFNA72	6p21.33	*SLC44A4*	Choline transporter plays a role in the choline–acetylcholine system.	Adulthood (3rd decade)	U-shaped	Progressive	HL is initially limited to middle frequencies but gradually progresses to all frequencies [104].
DFNA73	12q21.31	*PTPRQ*	Regulation of cellular proliferation and differentiation.	Childhood to adulthood (1st–3rd decade)	Sloping	Progressive	The degree of HL can range from mild to severe [105,106].
DFNA74	7p14.3	*PDE1C*	Proliferation of vascular smooth muscle cells and neointimal hyperplasia.	Adulthood (3rd decade)	Sloping	Progressive	HL gradually progresses from a mild to profound degree [107].
DFNA75	7q22.1	*TRRAP*	Important role in transcription and DNA repair.	Adulthood (2nd decade)	Sloping	Progressive	HL is initially limited to middle and high frequencies, but gradually involves all frequencies [108].
DFNA76	3q23	*PLS1*	Actin-bundling protein of the stereocilia.	Childhood to adulthood (1st–4th decade)	Sloping	Stable/ Progressive	HL tends to be more severe at higher frequencies, ranging from a mild to profound degree [109,110].
DFNA77	16p13.11	*ABCC1*	Transport various molecules across extra- and intra-cellular membranes.	Adulthood (2nd–3rd decade)	Sloping	Progressive	HL is initially limited to high frequencies but progresses to all frequencies by the 4th–5th decade of life [111].
DFNA78	5q23.3	*SLC12A2*	Membrane protein important in maintaining proper ionic balance and cell volume.	Congenital	Flat	Stable	HL is generally profound and may be associated with motor delay due to vestibular dysfunctions. Motor delay often resolves with age [112,113].
DFNA79	4q21.22	*SCD5*	Membrane protein of the endoplasmic reticulum that catalyzes the formation of monounsaturated fatty acids.	Adulthood (3rd-7th decade)	Sloping	Progressive	HL is generally milder in female patients [114].
DFNA80	18q11.1–q11.2	*GREB1L*	Predicted to be involved in retinoic acid signaling.	Congenital	Flat	Stable	HL is generally profound and associated with absent or malformed cochleae (incomplete partition type I) and eighth cranial nerves [115,116].
DFNA81	2p11.2	*ELMOD3*	GTPase-activating protein.	Adulthood (3rd decade)	Sloping	Progressive	HL is slowly progressive and ranges from a severe to profound degree [117].
DFNA82	3p25.3	*ATP2B2*	P-type primary ion transport ATPase.	Childhood to adulthood (1st–6th decade)	Sloping	Progressive	HL is rapidly progressive and may be associated with mild vestibular abnormalities [118].
DFNA83	5q13.2	*MAP1B*	Important for axonal growth and synapse maturation during brain development.	Adolescence to adulthood (2nd–3rd decade)	Sloping	Progressive	HL ranges from a mild to profound degree. Distortion product otoacoustic emissions (DPOAE) are usually present, indicating the normal function of outer hair cells [119].
DFNA84	13q34	*ATP11A*	P4-ATPase.	Congenital to adulthood (1st–3rd decade)	Sloping	Progressive	HL is slowly progressive and is characterized by intrafamilial variation in disease severity [120].
DFNA85	1p36.12	*USP48*	Involved in the processing of poly-ubiquitin precursors.	Childhood to adulthood (1st–3rd decade)	Flat	Progressive	HL may be asymmetric [121].
DFNA86	18p11.32	*THOC1*	Participatation in apoptotic pathways.	Adulthood (4th decade)	Sloping	Progressive	HL gradually progresses to all frequencies, reaching the severe-to-profound degree in the 7th/8th decades of life [122].
DFNA87	1q21.3	*PI4KB*	Involved in Golgi-to-plasma membrane trafficking.	Congenital	Flat	Progressive	HL is generally profound and associated with inner ear malformations, such as incomplete cochlea partition and enlarged vestibular aqueduct [123].
DFNA88	1p34.3	*EPHA10*	Mediators of cell–cell communication, regulating cell attachment, shape, and mobility in neuronal and epithelial cells.	Adulthood (3rd–4th decade)	Sloping	Progressive	HL gradually progresses to a profound degree [124].
DFNA89	4q22.2	*ATOH1*	Transcriptional regulator.	Congenital/Childhood (1st decade)	Flat	Progressive	Onset of HL is at birth or in early childhood [125].

HL = Hearing loss. * DFNA29, DFNA35, DFNA45, DFNA46, DFNA61, and DFNA62 are reserved by the HUGO Gene Nomenclature Committee (HGNC).

## Data Availability

Not applicable.
